# The effectiveness of a structured validated questionnaire to assess student perception with virtual pharmacy simulation in pharmacy practice experiential education

**DOI:** 10.1371/journal.pone.0314117

**Published:** 2024-11-21

**Authors:** Palanisamy Amirthalingam, Shahul Hameed Pakkir Mohamed, Vinoth Prabhu Veeramani, Mathar Mohideen Nagoor Thangam, Majed Falah Alanazi, Muralikrishnan Dhanasekaran, Vasudevan Mani, Mostafa A. Sayed Ali

**Affiliations:** 1 Faculty of Pharmacy, Department of Pharmacy Practice, University of Tabuk, Tabuk, Saudi Arabia; 2 Faculty of Applied Medical Sciences, Department of Health Rehabilitation Sciences, University of Tabuk, Tabuk, Saudi Arabia; 3 Saveetha Institute of Medical and Technical Sciences (Deemed to the University), Saveetha College of Physiotherapy, Chennai, Tamil Nadu, India; 4 Faculty of Nursing, Department of Medical Surgical Nursing, University of Tabuk, Tabuk, Kingdom of Saudi Arabia; 5 Faculty of Pharmacy, Pharm.D. Program, University of Tabuk, Tabuk, Saudi Arabia; 6 Department of Drug Discovery & Development, Harrison College of Pharmacy, Auburn University, Auburn, Alabama, United States of America; 7 Department of Pharmacology and Toxicology, College of Pharmacy, Qassim University, Buraydah, Saudi Arabia; 8 Faculty of Pharmacy, Department of Clinical Pharmacy, Assiut University, Assiut, Egypt; Qatar University College of Pharmacy, QATAR

## Abstract

**Background and objective:**

MyDispense is one of the virtual simulations that has already been established as a suitable alternative for live experiential education in the pharmacy curriculum. However, there are no structured validated questionnaires available to assess the students’ perception while integrating MyDispense with pharmacy practice experiential education. Therefore, the present study aimed to validate a structured questionnaire and use the questionnaire to assess the student perception of various pharmacy practice experiential education.

**Methods:**

Content and construct validity procedure was used to validate the questionnaire. Two hundred students consented to participate in validating the questionnaire. The validated questionnaire assessed the students’ perception of integrating MyDispense with Introductory Pharmacy Practice Experience 2 (IPPE2) and Advanced Pharmacy Practice Experience (APPE) courses. The questionnaire was structured with four domains which were: exercise, instructor, technical, and communication. Each domain carried five items; therefore, the whole questionnaire had 20 items that succeeded in content validity. In the survey, 121 fourth-year and 117 fifth-year Pharm.D. students volunteered to convey their perception of integrating MyDispense with IPPE 2 and APPE, respectively. The survey was conducted before and after the MyDispense exam in both the courses.

**Results:**

The Cronbach’s α and McDonald’s ω coefficients were > 0.8 in all four domains, indicating that the items related to the four domains have good internal consistency. In Exploratory Factor Analysis (EFA), two items were found to cross-load in the exercise domain and removed. Therefore, the EFA proposes 18 items for the confirmatory factor analysis (CFA). In CFA, five fit indices were found to be satisfactory, and this indicates construct was good enough to assess the student perception. In IPPE 2, the pre-test response, the students had significantly higher satisfaction (p < 0.05) with all five items related to the technical domain. In APPE, the students had a significantly (p < 0.05) higher perception of all the items related to the exercise and technical domain in the pre-test compared to the post-test. Therefore, the student’s pre-test feedback allowed the instructor to identify and make the necessary corrections in the exercises to improve the quality exercises.

**Conclusion:**

This study provides a validated 18-item questionnaire to assess the student perception of integrating MyDispense in experiential education. The integration of MyDispense in experiential education needs to be done carefully by assessing student perception.

## 1. Introduction

Pharmacy practice education usually involves traditional classroom lectures and training in pharmacy; however, technical advancement in recent years has directed the paradigm shift from the conventional classroom to computer-based technology education [1–3]. Pharmacy practice experience education is the best way to prepare the pharmacy student seeking a career as a pharmacist since it offers cognitive, technical, and decision-making skills needed for medication dispensing [4–7]. Pharmacy simulation is a valuable tool in pharmacy practice experience education for training pharmacy students by providing real-world experience [4,5]. However, pharmacy simulation was found to have numerous challenges, including extensive planning, highly expensive simulated pharmacy, and the requirement of many teaching staff for supervision [6]. In this context, virtual simulation has been widely accepted by pharmacy schools since it minimizes the workload of teaching staff, cost, and time while offering pharmacy practice experiences to students [2,5,7].

MyDispense is a digital education platform that enables students to practice the skills of a pharmacist, from novice to highly advanced, in a safe virtual environment that is web-based and highly accessible [7]. The students can learn from their mistakes due to the instant feedback MyDispense in a risk-free environment [8]. Previous studies have established that the MyDispense database helped assess the various student skills regarding dispensing, communication, decision-making, and problem-solving [7–9]. This platform was widely accepted by various pharmacy schools worldwide and implemented in various pharmacy practice experience courses [9]. Although MyDispense plays a vital role in pharmacy practice experience education, recent research addressed numerous barriers regarding implementation, including a lack of training for the staff members in building or updating cases, lack of realism, and challenges in the harmonization of drug nomenclature [10,11].

The implementation of MyDispense is varied among the institutions in terms of exercises and assessment criteria [5,12]. Therefore, assessing students’ perception of MyDispense is essential to ensure effective implementation in every institution. Previous studies already addressed the student perception of integrating MyDispense in various pharmacy courses [7,13–15]. In this context, to the best of our knowledge, structured validated questionnaires are yet to be established [4,7,9]. Therefore, the present study aimed to validate the pioneer multi-dimensional questionnaire for assessing student perception while integrating MyDispense with several pharmacy practice experiential education. Additionally, the study aimed to assess the student perception regarding MyDispense in various pharmacy practice experiential education by using the validated questionnaire.

## 2. Methods

### 2.1 Study design and ethical considerations

The study was conducted in two parts. First, it was planned to validate a 5-point multi-dimensional structured Likert-scale questionnaire using content and construct validity procedure. Second, the student perception was assessed with a validated questionnaire in a cross-sectional survey of integrating MyDispense among the various pharmacy practice experiential education. The student volunteers were recruited during the period from 01.03.2022 to 30.12.2023 to integrate MyDispense for the first time at the Faculty of Pharmacy, University of Tabuk, Saudi Arabia. The exercises include patient fact-finding, patient education, answering patient questions, prescription monitoring, labeling, and dispensing (Table 1). Introductory Pharmacy Practice Experience 2 (IPPE 2) and Advanced Pharmacy Practice Experience (APPE) courses were offered in the PharmD curriculum during the fourth and fifth years, respectively.

**Table 1:** Details of various tasks related to the exam in MyDispense.

Communication skills	Technical skills
**Patient fact-finding** Asking any five of the following (The type of questions depends on the course) Age Pregnancy details (if female) Symptoms/Diagnosis Medical and medication history (if any) Asking medication adherence (if any medication history) Social history (Smoking, alcohol, tobacco, illicit drug use, etc.) Known allergies Usage of over-the-counter medications/herbal medications	**Prescription monitoring** Any five items of the following (The items included depending on the course) Date Name of the doctor and license number Medications (Name, dose, quantity, etc.) Direction for use Signature of the physician Refill instructions Drug-Drug interactions
**Patient Education** Educating the patient on the following (Level of education depends on the course) Chief complaint/Diagnosis Dosage regimen Direction for use Possible side effects Non-pharmacological management Counseling to stop using smoking /tobacco/ alcohol/illicit drugs Medication adherence	**Labeling** Any five items of the following (The item selection depends on the course) Date Physician name/affiliation/license number Pharmacist name/affiliation/license number/signature Drug name/dose/frequency/route of administration/time of administration/quantity Direction for use Barcode scanning Refill details
**Answering patient questions** Student must answer the five questions raised by the patient (The level of the question depends on the course)	**Dispensing** All the following items Right patient Right drug Right dose Right quantity Right formulation

### 2.2. Details of exercise and its conductance in MyDispense

The exercises created in MyDispense were based on the course learning outcomes of the courses IPPE2 and APPE. A virtual patient appeared on the computer monitor and sought for his/her prescription to be filled. The exercises in IPPE 2 mainly aimed to test technical skills, including prescription monitoring, labeling, dispensing, and assessing communication skills. Meanwhile, the exercises in APPE also test the technical skills of labeling and dispensing skills; however, the main objective was to test the communication skills in patient fact-finding, patient education, and, importantly, answering patient questions regarding medications. The tutorial and formative assessment case details were attached as S1 File. IPPE2 and APPE courses were conducted for thirteen weeks in the first semester, and MyDispense was integrated for the first four weeks. The students were given a demonstration by the instructor regarding the MyDispense exercise and the rubrics for evaluation in detail in the first week, followed by a tutorial exercise that took place in the second week of the semester. A pre-test survey was conducted immediately after the completion of the tutorial exercise. A formative assessment in the third week, followed by a post-test survey conducted immediately. The actual summative assessment took place in the fourth week and the surveys were not permitted according to the University policy before or after the summative assessment. Students were posted in the pharmacy from the fifth to the thirteenth week for real-world experience. The MyDispense demonstration, tutorial, and formative assessment were carried out in the computer lab with internet facilities at the Faculty of Pharmacy, University of Tabuk.

### 2.3. Ethical approval and informed consent

The study was approved by the local research ethics committee from the University of Tabuk, Saudi Arabia (Reference number: UT-187-42-2022). Written informed consent was obtained from the student who volunteered to participate in the study. The students were given bonus points for participation in surveys.

### 2.4 Study participants

Firstly, two hundred students participated in validating the questionnaire. Then, 238 students responded to the validated questionnaire in a cross-sectional survey to address their perception of MyDispense integrated with IPPE2 (n = 121) and APPE (n = 117) courses. In IPPE 2, 50 male (41.32%) and 71 female (58.67%) students participated in the study, and 55 males (47%) and 62 females (53%) were included in the APPE group.

### 2.5 Components of questionnaire

The questionnaire was structured with four domains, including exercise, instructor, technical, and communication. Each domain has five items; hence, 20 items were included in the 5-point Likert scale questionnaire (Table 2). All four domains have been developed by consensus among the authors of this research based on the IPPE 2 and APPE courses’ learning outcomes, teaching strategies, and instruction methods. Three domains, including exercise, technical, and communication, were developed on the basis of IPPE 2 and APPE courses’ learning outcome domains, including knowledge, cognitive, and skill. The instructor domain consists of teaching strategies and instruction methods for the course specifications of IPPE2 and APPE. The students’ responses were recorded as strongly agree, agree, neutral, disagree, and strongly disagree, which reflects the scores 5,4,3,2 and 1, respectively.

**Table 2:** Reliability statistics and exploratory factor analysis of the questionnaire.

Domain	No	Items	Factor Loadings			
			Resources	Instructor	Technical	Communication
**Exercise**	1	The exercise was related to the course objective	**.757**	.200	.151	.232
	2	The details related to the exercise were clear to me	**.755**	.199	.088	.047
	3	I felt the exercise was relevant to the real-world practice	.466	.309	-.034	.522
	4	I believe that the given exercise might improve my patient care skills	.410	.380	.064	.826
	5	I am aware of the rubrics and assessment criteria for the exercise	**.654**	.211	.145	.306
**Instructor**	6	The instructor had a thorough knowledge of the exercise	.046	**.727**	.266	.064
	7	The instructor had adequate experience in MyDispense	.191	**.684**	.216	.070
	8	The instructor thoroughly demonstrated the MyDispense session	.100	**.794**	.237	.048
	9	The instructor encouraged me to complete the exercise successfully	.160	**.598**	.203	.112
	10	The instructor was available to clarify my doubts (if any) regarding the exercise	.039	**.591**	.283	.129
**Technical**	11	The prescription was clear and I had access to all patient information	.350	.071	**.656**	.010
	12	I felt that dispensing drugs was like a real-world experience	.141	.115	**.703**	.010
	13	I was able to pick up the correct medication from the rags for dispensing	.278	.181	**.718**	-.018
	14	I felt the labeling process and barcode scanning was like a real-world experience	.238	.201	**.836**	.024
	15	I was able to track and manage the time of the exercise efficiently	.127	.238	**.736**	.094
**Communication**	16	I was confident during the interaction with the virtual patient	.139	.048	.186	**.707**
	17	It seemed like a real-world experience when I engaged with a virtual patient	.172	.176	.192	**.838**
	18	The interaction with the virtual patient was useful to complete the session successfully	.203	.112	.187	**.794**
	19	I was able to educate the patient through MyDispense session	.118	.087	.107	**.855**
	20	I was able to answer the questions asked by the virtual patient	.172	.154	.158	**.859**
		Cronbach’s α	0.868	0.848	0.885	0.929
		McDonald’s ω	0.863	0.851	0.885	0.930
		Percentage of variance	23.574	19.444	15.878	6.162
		Cumulative percentage of variance	23.574	43.019	58.896	65.058

Items in the cell-shaded which indicate Factor lading < 0.5 or Items with cross-loading > 0.32.

### 2.6 Validation of questionnaire

#### 2.6.1 Content validity

Content validity was performed to evaluate how well the questionnaire covered the relevant parts of the construct [16]. The questionnaire was distributed to the five experts in pharmacy practice education outside the institution for content validity. Item-level content validity indexes (I-CVIs) and the averaging of scale-level content validity index (S-CVI/Ave) were included in the content validity. The scores of I-CVIs ≥ 0.78 and S-CVI/Ave ≥ 0.90 were considered excellent content validity [17].

#### 2.6.2 Construct validity

Cronbach’s α and McDonald’s ω coefficients were used to assess the internal consistency of the questionnaire. Cronbach’s α and McDonald’s ω coefficients of values > 0.9, > 0.7 to ≤ 9, and < 0.7 are considered excellent, good, and poor, respectively [18,19]. The model was constructed with four domains, each with five items initially. Exploratory factor analysis (EFA) was performed using maximum likelihood extraction with a Varimax rotation method [20]. Factor loading > 0.5 was considered an inclusion criterion to retain the items related to their factor, and cross-loaded items with > 0.32 were removed from the construct [21]. Bartlett’s test for sphericity of < 0.05 and Kaiser-Meyer-Olkin–Measuring Sampling Adequacy (KMO-MSA) value ≥ 0.7 were considered acceptable for sampling adequacy [22,23]. The threshold for the cumulative percentage of variance was 50.2% was considered [24]. The robust unweight least square estimation method was used in confirmatory factor analysis (CFA) [25]. According to the recommendations of Hair et al., 2010, more than three fit indices used to establish the model fitness were Chi-square to degrees of freedom ratio (χ2/df), root mean square error of approximation (RMSEA), standardized root mean square residual (SRMR), comparative fit index (CFI), and Tucker Lewis index [26]. χ2/df < 5, RMSEA ≤0.08, SRMR ≤ 0.08, CFI >0.9, and TLI >0.9 were considered a good model fit [27]. The average variance extracted (AVE) ≥ 0.5 and construct reliability for the latent factors (≥ 0.7) were considered for the desired convergent validity [20]. The squared correlation (SC) values of one construct with other constructs are less than the AVE of a specific construct and were considered for the desired discriminant validity [28,29].

### 2.7 Sample size calculation

For the construct validity procedure, the sample size calculation was based on a 1:5 ratio (number of items: participants) [20]. In the split-half method, the first 100 students were given an odd number of items, and the remaining 100 were given an even number of items in the questionnaire. Therefore, 200 students were recruited to validate the questionnaire. A convenient sampling method was used in the cross-sectional survey.

### 2.8 Statistical analysis

Statistical Package for the Social Sciences (SPSS version 25.0) was used to perform reliability statistics, factor analysis, and data analysis of the cross-sectional survey. The Mean (Standard Deviation) value of student perception was compared between the pre-test and post-test by using the Wilcoxon signed rank test. P < 0.05 was considered statistically significant.

## 3. Results

### 3.1 Validation

The I-CVIs for the relevancy of the questionnaire ranged from 0.8 to 1, and the S-CVI/Ave was >0.9. Hence, I-CVIs and S-CVI/Ave demonstrated that the 20-item questionnaire underlying four factors has excellent content validity. The Cronbach’s α and McDonald’s ω coefficients were > 0.8 in all four factors, indicating that the items related to the four factors have good internal consistency (Table 2). The sample adequacy was confirmed by the KMO-MSA (0.896) and Bartlett’s test for sphericity (p < 0.001). The total cumulative percentage of variance (65.05%) indicates that the proportion of variance explained by the factors was satisfactory. A couple of items with cross-loading values > 0.32 in the exercise were removed from the questionnaire (Table 2). Hence, an 18-item questionnaire was proposed by EFA, and the same was investigated in CFA. The four-factor questionnaire construct was found to have acceptable fit indices, including χ2/df (2.16), RMSEA (0.076), SRMR (0.045), CFI (0.938), and TLI (0.927) (Table 3). The average variance extracted (AVE) of each factor was > 0.5, indicating the convergent validity of the construct (Table 4). The discriminant validity was determined since the AVE of each factor was more than the squared correlations (SC) with the other factors (Table 4). Therefore, an 18-item questionnaire with four factors was constructed successfully to assess the students’ perception regarding virtual simulation.

**Table 3:** Model fit indices of CFA.

	CFI	TLI	SRMR	RMSEA	90% confidence interval		χ^2^	df	χ^2^/ df
					Lower	Upper			
Observed	0.938	0.927	0.0455	0.0763	0.0640	0.0885	279	129	2.16
Reference	> 0.9	> 0.9	< 0.08	< 0.08	-	-	-	-	< 6

CFI: Comparative Fit Index; TLI: Tucker Lewis Index; SRMR: Standardized Root Mean Square Residual; RMSEA: Mean Square Error of Approximation; χ^2^: Chi-Square; df: Degrees of Freedom.

**Table 4:** Convergent validity and discriminant validity of CFA.

	**Composite reliability**	**Resources**	**Instructor**	**Technical**	**Communication**
**Resources**	0.849	**0.533**			
**Instructor**	0.841	(0.390)	**0.517**		
**Technical**	0.891	(0.135)	(0.283)	**0.620**	
**Communication**	0.936	(0.265)	(0.125)	(0.158)	**0.745**

Average variance extracted (AVE) in bold letters; Squared correlations (SC) mentioned in bracket.

AVE > 0.5 and AVE > SC values (for the corresponding factors) determine the convergent and discriminant validity.

### 3.2 Survey

The students were asked to complete the questionnaire before and after the test in a virtual simulation for the courses IPPE 2 and APPE. Overall, the students’ perceptions were good in the pre-and post-tests for both IPPE 2 and APPE courses (Tables 5 and 6). In IPPE 2, the students’ perception of pre-and post-tests had no significant difference regarding exercise, instructor, and communication domains. However, in the pre-test response, the students had significantly higher satisfaction (p < 0.05) with all five items related to the technical domain. In APPE, the students had a significantly higher perception of all the items related to the exercise and technical domain in the pre-test compared to the post-test. Satisfaction was significantly higher in the pre-test regarding three items of the instructor domain about the instructor having adequate experience in MyDispense (p = 0.015), the instructor encouraging me to complete the exercise successfully (p = 0.002), and the instructor being available to clarify my doubts. Three items in the communication domain had significantly higher perceptions in the pre-test, including "I was confident during the interaction with the virtual patient" (p = 0.000), "It seemed like a real-world experience when I engaged with a virtual patient" (p = 0.000), and "The interaction with the virtual patient was useful to complete the session successfully" (p = 0.000).

**Table 5:** Student perception with a virtual siulation of the IPPE 2 course (n = 121).

No	Items	Mean (SD)		Wilcoxon signed-rank test	
		Pre-test	Post-test	z	p
1	The exercise was related to the course objective	4.13 (1.35)	4.37 (1.06)	-0.849	0.396
2	The details related to the exercise were clear to me	4.30 (1.21)	4.50 (0.83)	-0.427	0.669
3	I am aware of the rubrics and assessment criteria for the exercise	4.48 (1.06)	4.58 (0.72)	-0.600	0.548
4	The instructor had a thorough knowledge of the exercise	4.45 (1.02)	4.50 (0.72)	-0.720	0.472
5	The instructor had adequate experience in MyDispense	4.49 (1.04)	4.57 (0.78)	-0.339	0.735
6	The instructor thoroughly demonstrated the MyDispense session	4.60 (0.75)	4.55 (0.73)	-0.735	0.462
7	The instructor encouraged me to complete the exercise successfully	4.55 (0.88)	4.51 (0.83)	-0.827	0.408
8	The instructor was available to clarify my doubts (if any) regarding the exercise	4.43 (1.11)	4.41 (0.90)	-1.397	0.162
9	The prescription was clear and I had access to all patient information	4.37 (0.86)	4.18 (0.74)	-2.616	**0.009**
10	I felt that dispensing drugs was like a real-world experience	3.92 (1.19)	3.64 (1.06)	-2.232	**0.026**
11	I was able to pick up the correct medication from the rags for dispensing	3.91 (1.21)	3.61 (1.05)	-2.317	**0.021**
12	I felt the labeling process and barcode scanning was like a real-world experience	4.43 (0.80)	4.21 (0.76)	-2.862	**0.004**
13	I was able to track and manage the time of the exercise efficiently	4.24 (0.99)	3.89 (0.94)	-3.073	**0.002**
14	I was confident during the interaction with the virtual patient	3.94 (1.28)	3.87 (0.96)	-1.653	0.098
15	It seemed like a real-world experience when I engaged with a virtual patient	3.93 (1.34)	3.74 (1.21)	-1.753	0.080
16	The interaction with the virtual patient was useful to complete the session successfully	3.79 (1.14)	3.73 (0.91)	-1.219	0.223
17	I was able to educate the patient through MyDispense session	3.58 (1.39)	3.60 (1.07)	-0.239	0.811
18	I was able to answer the questions asked by the virtual patient	4.06 (1.19)	3.90 (1.08)	-1.941	0.052

**Table 6:** Student perception with a virtual simulation of the APPE course (n = 117).

No	Items	Mean (SD)		Wilcoxon signed-rank test	
		Pre-test	Post-test	z	p
1	The exercise was related to the course objective	4.68 (0.82)	4.38 (0.99)	-3.164	**0.002**
2	The details related to the exercise were clear to me	4.60 (1.00)	4.44 (0.77)	-3.153	**0.002**
3	I am aware of the rubrics and assessment criteria for the exercise	4.62 (0.96)	4.46 (0.95)	-2.389	**0.017**
4	The instructor had a thorough knowledge of the exercise	4.22 (1.20)	4.59 (0.66)	-1.942	0.052
5	The instructor had adequate experience in MyDispense	4.68 (0.87)	4.54 (0.76)	-2.441	**0.015**
6	The instructor thoroughly demonstrated the MyDispense session	4.21 (1.31)	4.50 (0.75)	-0.585	0.559
7	The instructor encouraged me to complete the exercise successfully	4.34 (1.25)	4.28 (0.73)	-3.098	**0.002**
8	The instructor was available to clarify my doubts (if any) regarding the exercise	4.33 (1.29)	4.00 (0.96)	-4.074	**0.000**
9	The prescription was clear and I had access to all patient information	4.64 (0.89)	3.92 (0.98)	-6.483	**0.000**
10	I felt that dispensing drugs was like a real-world experience	4.57 (0.99)	4.17 (0.80)	-5.550	**0.000**
11	I was able to pick up the correct medication from the rags for dispensing	4.50 (0.91)	3.85 (0.81)	-6.582	**0.000**
12	I felt the labeling process and barcode scanning was like a real-world experience	4.38 (1.01)	3.63 (0.92)	-6.169	**0.000**
13	I was able to track and manage the time of the exercise efficiently	4.42 (0.99)	3.85 (0.98)	-5.420	**0.000**
14	I was confident during the interaction with the virtual patient	4.39 (1.07)	3.74 (0.85)	-6.220	**0.000**
15	It seemed like a real-world experience when I engaged with a virtual patient	4.48 (1.05)	3.47 (1.00)	-7.758	**0.000**
16	The interaction with the virtual patient was useful to complete the session successfully	4.51 (0.97)	3.70 (0.84)	-8.065	**0.000**
17	I was able to educate the patient through MyDispense session	4.38 (1.06)	4.20 (1.04)	-1.918	0.055
18	I was able to answer the questions asked by the virtual patient	4.23 (1.29)	4.26 (0.99)	-1.313	0.189

## 4. Discussion

The study validated an 18-item structured questionnaire to assess the student perception of virtual pharmacy. It was already well established that MyDispense supports academicians to enhance student learning, increase academic and practical knowledge, and develop essential skills needed to become a pharmacist [9,30,31]. The Pharmacy students’ career option preference as a pharmacist was strongly influenced by their satisfaction with the activities in the Pharmacy curriculum [32]. Therefore, it is important to obtain students’ perceptions periodically to update the activities in the curriculum. Previous studies addressed the student perception with the questionnaire related to the learning, technical, and communication aspects in terms of virtual patients [4,13,15,33]. In addition, our questionnaire addresses the students’ perceptions about the suitability of exercise for the course and clarity of exercise and assessment criteria. Also, the questionnaire can know the student’s perception regarding their instructor in various aspects, including knowledge of the exercise, experience in MyDispense, demonstration skills, motivation of students, and availability to sort out any difficulties. Successful implementation of the virtual simulation exercise depends on the resources available in the institution [34]. Integrating virtual pharmacy simulation among experiential education needs a careful selection of exercises and strategic implementation of them within the courses [30]. Student perception of instructors is strongly related to their satisfaction with online learning, and motivation of students toward virtual platforms will help to achieve the learning outcomes [35–37]. Hopefully, the additional domains in the questionnaire will help the academicians improve the quality of creating and implementing the exercises and instructional approach through the feedback from the students.

The questionnaire was implemented in pre-test and post-test in both IPPE 2 and APPE. Overall, the student’s perception was found to be good regarding integrating MyDispense with IPPE 2 and APPE in terms of all the domains in the questionnaire. In IPPE2, the students found difficulty regarding technical aspects during the test, reflected in their post-test responses. To the best of our knowledge, there are no reports of psychological or physiological factors, if any, deteriorating student performance while appearing with MyDispense for the first time. However, previous researchers reported that virtual simulation reduces anxiety among occupational therapy students [38]; however, another author published a conflicting report that virtual simulation was capable of causing psychological and physiological stress among medical and nursing students [39]. This study warrants future studies to investigate the correlation between the level of stress and anxiety with performance while integrating MyDispense in introductory experiential education. The exercises in the tutorial and formative assessment might have differences in complexity level (S1 File). In this regard, the complexity of the exercise could be the potential confounding factor for the significantly lower perception of the students in the post-test survey. Therefore, the academicians need to pay more attention to maintain the complexity level of exercise in both tutorial and evaluation.

The usefulness of MyDispense in improving communication skills has already been established regarding comprehensive patient-fact findings, and it also facilitates the students to prepare the counseling points in various practice set-ups [9]. The exercises in APPE mainly focussed on testing the skills related to the communication aspects followed by technical skills. The student perception related to all the items in the exercise and three items related to the instructor domain were significantly lower in the post-test survey. This feedback from the students will allow the academicians to improve the quality of exercises and the involvement of instructors [30,35–37]. The students highly perceived the MyDispense pre-test in all the items of the technical domain and three items in communication aspects. It might be due to the psychological or physiological stress associated with the students during the exam [39]. The post-test student responses imply that the student needs more motivation to answer patient questions and dispense multiple medications in APPE at a given time. Henceforth, the academicians can able to improve the quality of exercises and improve their participation to enhance the student’s face-to-face real-world experience in terms of handling complex situations in pharmacy practice [32,40].

### 4.1 Strength and limitations

The study has come up with a new 18-item validated questionnaire for the first time, and the questionnaire can be implemented in both introductory and advanced experiential education. However, the study has the following limitations: 1. A limited sample size may be unable to judge all the aspects of exercises, instructor, technical, and communication, 2. The study was conducted in one site, and the results cannot be generalized. 3. The questionnaire was validated by acquiring students from a single institution; it could have been obtained from students of different Universities, and 4. Both pre-test and post-test surveys took place after only one exercise might not be enough to reflect student satisfaction accurately.

## 5. Conclusion

This study supplies a validated 18-item questionnaire to assess the student perception of the integration of MyDispense with introductory and advanced experiential education. This questionnaire can help the academicians to enhance the quality of exercises and instructors by acquiring the student’s perceptions. The complexity of exercises reflects the student satisfaction; hence, the instructors need to be well-prepared with good-quality exercises and also need to encourage the students to perform well while integrating MyDispense with advanced-level experiential education.

## Supporting information

S1 File Case details. (DOCX)
