# Supplementary material for: The effectiveness of a structured validated questionnaire to assess student perception with virtual pharmacy simulation in pharmacy practice experiential education
Source: PLoS One. 2024 Nov 21;19(11):e0314117. doi: 10.1371/journal.pone.0314117 (PMC11581236; doi:10.1371/journal.pone.0314117)
Supplement: S1 File — (DOCX) [file pone.0314117.s001.docx]

**Supplementary File 1**

**Case details for IPPE 2**

**Tutorial exercise**

Mrs. TS is waiting for you to get the medications on his prescription. Recently, she was diagnosed with hypothyroidism.

Rx

Levothyroxine sodium 75 mcg PO once a day

**Exercise for formative assessment**

Mrs. MR needs her medication to be dispensed for her prescription; she has a history of hypertension and dyslipidemia.

Rx

Amlodipine 10 mg OD PO in the morning

Atenolol 50 mg OD PO at bedtime

Atorvastatin 10 mg OD PO at bed time

**The student must complete the following tasks**

1. Patient fact-finding

- Ask questions to the virtual patient relevant to the case scenario

1. Prescription monitoring

- Check the date of prescription, prescriber details, patient details, medication details, and signature of the physician
- Do not dispense if any of the above information is wrong

1. Arrange the medications and label them
2. Dispense the medications

**Case details for APPE**

**Tutorial exercise**

Mr. SM was recently diagnosed with major depressive disorder, and he is seeking medications for his prescription.

Rx

Escitalopram 20 mg OD PO before bedtime

**Exercise for formative assessment**

Mrs. SM is seeking medications for her prescription, who was recently diagnosed with bipolar disorder.

Rx

1. Lithicarb 250 mg tablets twice daily
2. Tegretol 200 mg tablets twice daily

**The student must complete the following tasks**

1. Patient fact-finding
2. Label and dispense the medications
3. Educate the patient (chief complaint, dosage regimen, possible side effects, non-pharmacological approach, and the importance of medication adherence.)
4. Answer patient questions (At least five questions)

Note: Students can use <https://www.uptodate.com/>, available in the Saudi Digital Library (https://myut.ut.edu.sa/), to find relevant information about the case.
